# Integrating Liquid Biopsy and Radiomics to Monitor Clonal Heterogeneity of EGFR-Positive Non-Small Cell Lung Cancer

**DOI:** 10.3389/fonc.2020.593831

**Published:** 2020-12-16

**Authors:** Federico Cucchiara, Marzia Del Re, Simona Valleggi, Chiara Romei, Iacopo Petrini, Maurizio Lucchesi, Stefania Crucitta, Eleonora Rofi, Annalisa De Liperi, Antonio Chella, Antonio Russo, Romano Danesi

**Affiliations:** ^1^ Clinical Pharmacology and Pharmacogenetics Unit, Department of Clinical and Experimental Medicine, University of Pisa, Pisa, Italy; ^2^ Pneumology Unit, Cardiovascular and Thoracic Department, Azienda Ospedaliero-Universitaria Pisana, Pisa, Italy; ^3^ Radiology Unit 2, Department of Diagnostics and Imaging, Azienda Ospedaliero-Universitaria Pisana, Pisa, Italy; ^4^ Department of Translational Research and New Technologies in Medicine and Surgery, University of Pisa, Pisa, Italy; ^5^ Section of Medical Oncology, Department of Surgical, Oncological and Stomatological Sciences, University of Palermo, Palermo, Italy

**Keywords:** non-small cell lung cancer, EGFR, liquid biopsy, cell free DNA, radiomics, tyrosine kinase inhibitors, precision medicine

## Abstract

**Background:**

EGFR-positive Non-small Cell Lung Cancer (NSCLC) is a dynamic entity and tumor progression and resistance to tyrosine kinase inhibitors (TKIs) arise from the accumulation, over time and across different disease sites, of subclonal genetic mutations. For instance, the occurrence of EGFR T790M is associated with resistance to gefitinib, erlotinib, and afatinib, while EGFR C797S causes osimertinib to lose activity. Sensitive technologies as radiomics and liquid biopsy have great potential to monitor tumor heterogeneity since they are both minimally invasive, easy to perform, and can be repeated over patient’s follow-up, enabling the extraction of valuable information. Yet, to date, there are no reported cases associating liquid biopsy and radiomics during treatment.

**Case presentation:**

In this case series, seven patients with metastatic EGFR-positive NSCLC have been monitored during target therapy. Plasma-derived cell free DNA (cfDNA) was analyzed by a digital droplet PCR (ddPCR), while radiomic analyses were performed using the validated LifeX® software on computed tomography (CT)-images. The dynamics of EGFR mutations in cfDNA was compared with that of radiomic features. Then, for each EGFR mutation, a radiomic signature was defines as the sum of the most predictive features, weighted by their corresponding regression coefficients for the least absolute shrinkage and selection operator (LASSO) model. The receiver operating characteristic (ROC) curves were computed to estimate their diagnostic performance. The signatures achieved promising performance on predicting the presence of EGFR mutations (R^2^ = 0.447, p <0.001 EGFR activating mutations R^2^ = 0.301, p = 0.003 for T790M; and R^2^ = 0.354, p = 0.001 for activating plus resistance mutations), confirmed by ROC analysis.

**Conclusion:**

To our knowledge, these are the first cases to highlight a potentially promising strategy to detect clonal heterogeneity and ultimately identify patients at risk of progression during treatment. Together, radiomics and liquid biopsy could detect the appearance of new mutations and therefore suggest new therapeutic management.

**Graphical Abstract:**
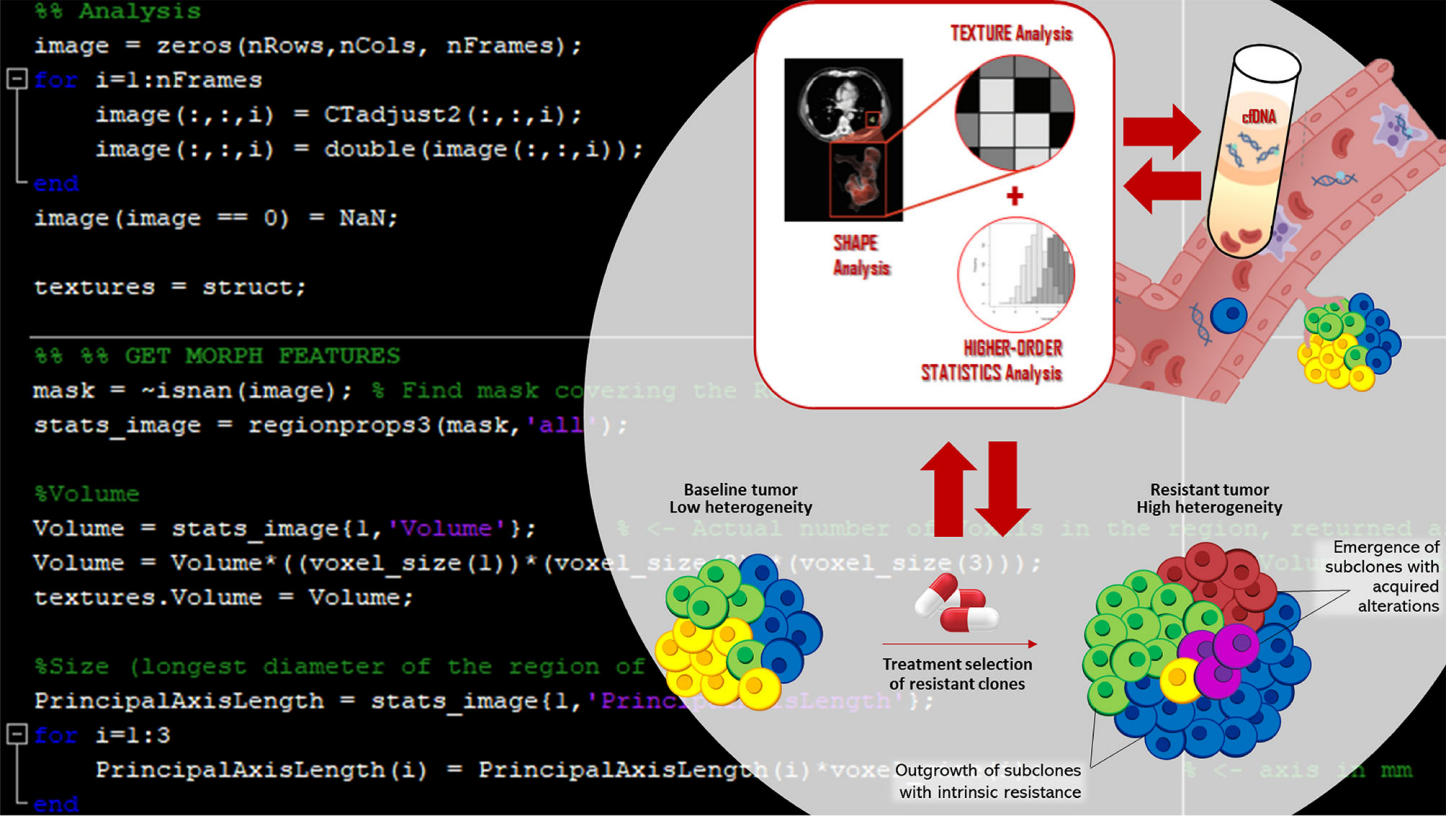


## Introduction

Tyrosine kinases inhibitors (TKIs), such as gefitinib, erlotinib, afatinib or osimertinib, are the first-line treatments in patients with advanced NSCLC and activating EGFR mutation (1–4) since they improved progression-free survival (PFS) compared with conventional chemotherapy (5). Nevertheless, NSCLC is a dynamic entity, and tumor progression and resistance to treatment arise from the accumulation of independent genetic mutations in subclones, over time and across different disease sites, thereby resulting in temporal and spatial heterogeneity (6). Moreover, treatment exerts selective pressure on cancer cells, and only those bearing either primary or secondary resistance mutations will survive (7–9).

The concept of a single-site biopsy to monitor disease dynamics is practically unfeasible since it is invasive and may result in underestimation of heterogeneity (10). Instead, liquid biopsy—allowing the analysis of cell-free DNA (cfDNA) (11)—better reflects the mutational status from the overall sites of disease (12), being able to identify emerging sub-clones responsible for treatment resistance (13).

Besides, radiomics has emerged as a novel field of research (14), dealing with the extraction and analysis of specific features from diagnostic images (15), and potentially reflecting the pathophysiological processes and the heterogeneity of tumors genetics (16). Recent data has shown that also texture analysis of radiological images can identify NSCLCs bearing EGFR mutations (17, 18).

The combined approach of radiomics and liquid biopsy has the potential to understand the dynamics of molecular lesions, supporting clinical decision-making.

To date, no reports correlate the dynamics of EGFR mutations in cfDNA with that of radiomic features. The present study aimed to assess such correlation in a case series of seven patients with EGFR mutant NSCLC, and to build a multi-parametric signature of clonal heterogeneity.

## Patients

The study retrospectively matched clinical, molecular and imaging databases of seven patients with histologically proven EGFR-positive NSCLC (exon 19 deletion [ex19del], exon 21 [L858R], or other mutations [i.e. L861Q]), and candidate to a first/second or third-generation EGFR-TKIs. Enrolled patients underwent blood sampling 1) before the first dose of TKI (baseline), 2) every two months, 3) and at each of the instrumental (i.e. imaging) disease re-evaluation throughout the follow-up. Complete (CR) and partial response (PR), disease stabilization (SD) and disease progression (PD) were defined following RECIST (v. 1.1) criteria. CT scans were collected at baseline and every 3–6 months as per clinical practice (19) and then used for radiomic analysis. The interval between follow-up medical visits (3 vs. 6 months) was based on clinical decision-making on an individual basis (19). Clinical data were collected from medical records. A written consent form was obtained from all patients. The study was approved by the institutional ethics committee of University Hospital, Pisa, Italy (protocol 5625/2015), and performed in accordance with the provisions established by the Helsinki Declaration.

## Methods

### cfDNA Extraction and Analysis

cfDNA was extracted from 3 ml of plasma using the QIAmp Circulating Nucleic Acid kit (Qiagen®, Hilden, Germany) and then eluted in 100 μl of the buffer, as previously described (20). EGFR mutations (ex19del, L858R, T790M, and C797S) were investigated by digital droplet polymerase chain reaction (ddPCR) using the ddPCR Mutation Assay (BioRad®, Hercules, CA). A fluorescence intensity threshold of 3,000 was set as a cut-off point; the sample was considered as mutant positive when at least one droplet was above the threshold level. The number of mutant alleles was reported as copies/ml.

### CT Segmentation and Extraction of Radiomic Features

Images were extracted from multiple non-contrast material-enhanced thoraco-abdominal computed tomography (CT)-scans (SIEMENS CT Sensation 64®; kilovoltage = 120 KV and exposure = 165 mAs; CT slice = 1.5 mm) (21). All CT examinations were reconstructed using B30f kernel (22). The radiomic analysis was performed by one author, using the validated LifeX® software (LifeX®, IMIV, CEA, Inserm, CNRS, Orsay, France) (23–25), after appropriate manual segmentation of the volumes of interest (VOIs; i.e. the lesions). Thirty-six radiomic features including three shapes, two gray-level histogram, six gray-level co-occurrence matrix (GLCM), 11 gray-level run lengths matrix (GLRLM), three Neighborhood Grey-Level Different Matrix (NGLDM) and 11 Grey-Level Zone-Length Matrix (GLZLM) features, were computed.

### Selection of Radiomic Features and Data Analysis

Each patient had a longitudinal dataset of several scans to match with respective temporally linked liquid biopsy data. To calculate to which extent the variation between the radiomic features is correlated to EGFR mutation status, a logistic least absolute shrinkage and selection operator (LASSO) regression model adopting a 27-fold Monte Carlo cross-validation was applied and executed in Matlab R2019a (MatLab® software, The Math Works Inc., Natick, MA) (26, 27). The LASSO logistic model was used to reduce the number of radiomic features and estimate the maximum-likelihood fitted regression coefficients for the remaining ones. The LASSO computation was performed to assess the radiomic features about the copies/ml of EGFR activating mutations (ex19del/L858R), as well as about the emergence of resistance mutations (T790M and C797S) and the total copies/ml (ex19del/L858R together with T790M and C797S) occurring in EGFR in patients progressed to TKI treatments. Selected radiomic features are reported in **Supplemental Table 1** in the online version. Then, radiomic signatures were calculated as the sum of the selected features weighted by their corresponding regression coefficients for the LASSO models. The receiver operating characteristic (ROC) curve analysis was computed to estimate the diagnostic performance of such signatures and select the optimal thresholds.

Moreover, Kendall’s correlation coefficient (tau-b, τb) was calculated to determine the strength of the association between changes in radiomic features and changes in matched liquid biopsy-derived data, over time (28).

Differences were considered significant at p <0.05. Statistical analysis was performed using the open-source statistical language R (R Foundation for Statistical Computing, Vienna, Austria) through the free and open statistical software program JAMOVI® (Version 1.1.9; retrieved from https://www.jamovi.org).

## Results

**Figure 1:**
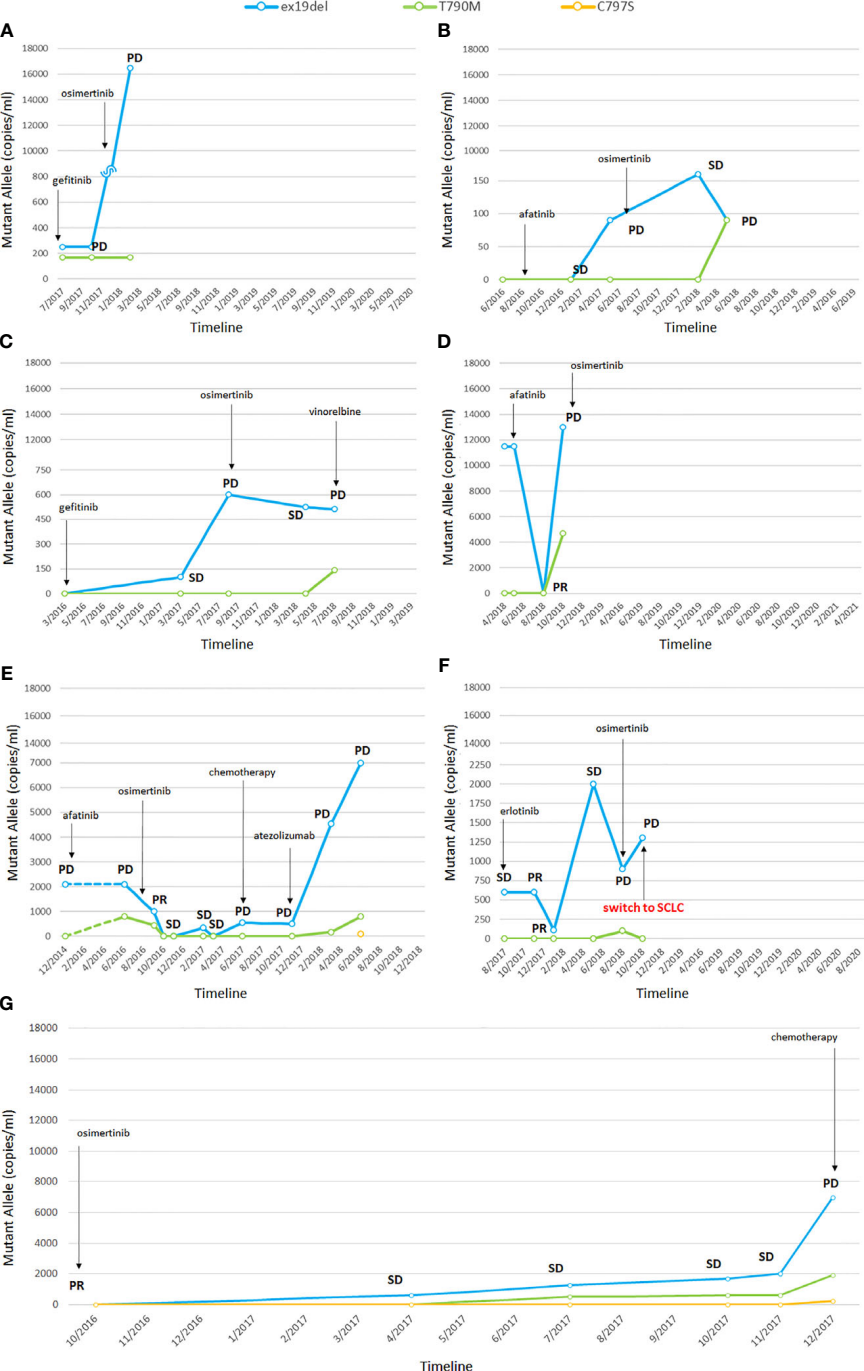
Changes in EGFR mutations detected by liquid biopsy **(A–G)**. In one patient the tumor transformed into small cell lung cancer (SCLC) **(F)**. PR, partial response; SD, stable disease; PD, progressive disease; SCLC, small cell lung cancer. The numbers before the year indicate the months while the letters **(A–G)** refer to single patients.

Five patients presented the ex19del activating mutation at diagnosis and two were carriers of the L858R. Three patients were treated with afatinib, two with erlotinib, and two with gefitinib as first-line TKI. Clinical characteristics are summarized in **Supplemental Table 2** in the online version, while plasma monitoring for each of them is reported in **Figure 1**. Overall, at baseline, the median activating EGFR copies/ml was higher than T790M and was often related to disease control, whereas the T790M amount was not. At disease progression, T790M was detected in plasma and/or tissue in all patients; therefore, osimertinib treatment was started, except in one patient, since the drug was not yet available (**Figure 1G**). In two patients disease progression occurred due to C797S mutation, in addition to ex19del and T790M.

The dynamics of EGFR mutations were significantly associated to specific changes in radiomic features over time (p <0.05; **Supplemental Table 3** in the online version). Radiomic features selected at least once in the LASSO models with respect to the number of copies/ml of mutant EGFR L858R/ex19del were combined in a signature (R).

R=∑radiomic signature*regression coefficients

**Figure 2:**
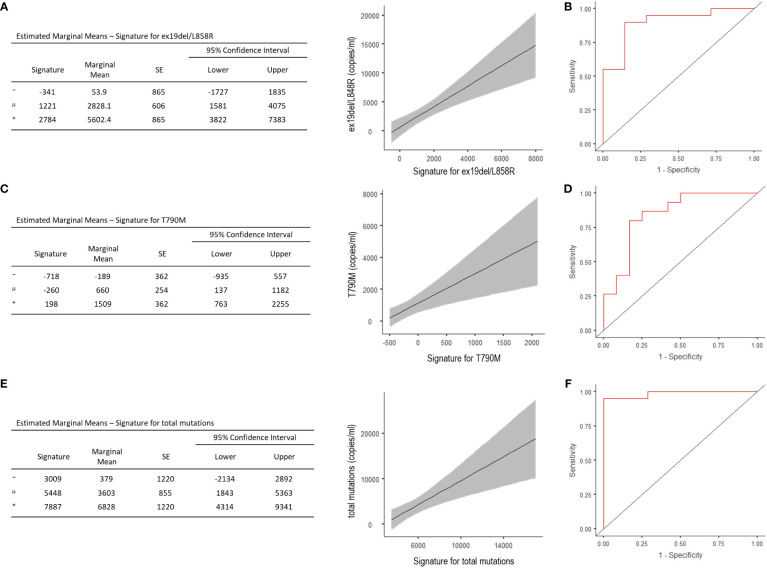
Estimated Marginal Means and ROC analysis for radiomic signature concerning ex19del/L858R **(A, B)**, T790M **(C, D)**, and total copies/ml (i.e. ex19del/L858R together with T790M and C797S) **(E, F)**. ⁻, mean-1SD; μ, mean; ⁺, mean+1STD.

The signature evidenced good capability—with acceptable representativeness—in predicting the number of copies/ml of the activating EGFR (R^2^ = 0.447, Akaike’s Information Criteria (AIC) = 515, p <0.001) (**Figure 2A**), and the optimal cut-point estimated from the ROC curve showed 88.9% accuracy, 90% sensitivity, and 85.7% specificity, with the area under the curve (AUC) of 0.90 (**Figure 2B**). Signatures and their predicting representativeness were also evaluated with respect to the copies/ml of T790M mutation and the total copies/ml of mutations in patients progressed to TKIs. The model predicting the T790M copies/ml showed R^2^ = 0.301 and AIC = 468 (p = 0.003), with 81.5% accuracy, 80% sensitivity and 83.3% specificity of the optimal threshold, and AUC of 0.84 (**Figures 2C, D**). The model predicting the total copies/ml of all mutations displayed R^2^ = 0.354 and AIC = 534 (p = 0.001), with 96.3% accuracy, 95% sensitivity, and 100% specificity of the optimal threshold, with AUC of 0.98 (**Figures 2E, F**); no correlation emerged for C797S.

## Discussion

To date, several works studied the potential of radiomics in the non-invasive prediction of EGFR mutational status and showed promising results (18, 24, 29–32). However, none of them addressed the subclonal heterogeneity that occurs asynchronously. In this study, we endeavored to highlight the great potential of integrating radiomics and liquid biopsy, as both are minimally invasive, easy to perform, and can be repeated over patients’ follow-up visits.

We did it by incorporating multiple radiomic functions into a signature (R) that could reliably predict the EGFR mutation status during treatment, and demonstrating significant correlations between radiomics and liquid biopsy data.

Of note, sphericity of lung lesions decreased with the increase of T790M copies/ml and, more generally, with the total copies/ml of mutant alleles, highlighting the association between spherical disproportion and neoplastic progression, aggressiveness and resistance to therapy (33). Besides, copies/ml of the T790M mutation were directly correlated with GLCM dissimilarity (a measure of local intensity variation of the voxel gray levels), and direct relationships also emerged between ex19del/L858R copies/ml, GLCM energy and contrast (**Supplemental Table 3**). The GLCM energy is a characteristic that describes the order status of the system and refers to the uniformity of the gray level between voxel pairs. The GLCM contrast highlights how many nearby sub-areas of heterogeneity differ within each lesion.

Interestingly, we found no significant correspondence between tumor volume (ml) and mutational status (**Supplemental Table 3**). Our results are consistent with those from Park et al. (34) and Lee et al. (35), testing the stability and reliability of radiomic features to evaluate tumor heterogeneity. Notably, Lee and collaborators (35), by studying the variability of radiomics features and their relationship with tumor size and shape upon 260 lung nodules, found that only a few features—including spherical disproportion and dissimilarity—showed high reproducibility in correlation with nodule status. This is probably because radiomics in lung cancer is different from in other oncology fields. Lung cancer resides in an environment rich with air, while other cancers primarily consist of soft tissue and reside in the interstitium (36). More than the usual volume changes, tumor progression is associated with shape and density changes from ground-glass opacity (GGO) to solid component (37–39). Thus, radiomics in the lung should jointly consider the tumor core geometry along with textural changes to properly model lung cancers. Nevertheless, reproducibility studies are lacking, and more evidences are needed to provide suggestions for future lung radiomics investigations.

There was also no significant correlation between changes in radiomic characteristics and C797S dynamics, probably due to the low sample size. However, the landscape of mechanisms of resistance dramatically changes considering osimertinib, and future studies to better investigate radiomic changes correlating with C797S dynamics will be needed (40). The appearance of the EGFR C797S mutation accounts for 6–10% after osimertinib as first line and 10–26% as second line (41). Furthermore, co-occurring with T790M has potential implications for treatment: when C797S and T790M occur on the same allele (*cis*), no response to EGFR TKIs alone or in combination can be expected, while the C797S in trans with the T790M mutation confers sensitivity to a combination of first/third-generation drugs (42–44).

Our results took advantage of consistently examining a few patients over a period, and of correlating changes in their radiomic features with the respective dynamics of EGFR mutations in cfDNA. The resulting signatures showed a good capability—with acceptable representativeness—in predicting the tumor mutational status. Unfortunately, given the low number of subjects, we found no radiomic signature that can be reliably associated with clinical outcomes, but we plan to look for it in the future. Future larger prospective clinical trials will also need to validate these findings and give us the chance to look for new resistance signatures, such as the one related to SCLC transformation, which is an important potential mechanism of resistance for to first/second and third-generation EGFR-TKIs (8), but to date, only a new tissue biopsy could allow to find it. Clinicians may consider using the signature as a new supporting tool, in accordance with their experience and judgment.

Liquid biopsy and radiomics have both advantages and drawbacks making them complementary methods. Although they are appealing options at progression, to track mechanisms of resistance (40, 45–48), there are still too few laboratory applications for liquid biopsy, and molecular protocols need to be standardized. Furthermore, there are difficulties in detection, and extremely sensitive and specific analytical methods are required to deal with small quantities of easily degradable materials. Lastly, it is still unclear whether liquid biopsy provides a representative sampling of all genetic clones or whether there is a propensity for specific subpopulations within the intra-tumor heterogeneity (49). Similarly, radiation, dearth of standardization for image acquisition, computational approaches and feature selection, as well as the black-box problem (i.e. non-interpretable advanced machine-learning algorithms that work like black boxes, hindering clinical translation), limit the use of radiomics, which should be considered as an indirect and non-detailed quantification of the underlying biological processes. Therefore, to strengthen the trustworthiness of the results, radiomics-based genotype predictions could be compared with information from liquid biopsy (16), over time. A combination of these two minimally invasive strategies, together with cutting-edge data analysis strategies, could be more valuable and reliable than their independent use and may help decode tumor information regarding the type, aggressiveness, progression, and response to treatment (29, 50). A study from the University of Oklahoma reported that while radiomics and genomics models were capable of predicting survival, accuracy significantly improved when both data were combined (51). Besides, while it is possible to avoid unnecessary radiation by using liquid biopsy, on the other hand, we can use radiomics to refine liquid biopsy results and provide a full-field analysis of patient’s lesions in virtually real-time response. Both techniques, providing a new instrumental and therefore objective diagnostic support, are able to reduce the need for invasive (and often difficult to perform) biopsies and favor an approach that promptly suggests a change in treatment strategy over the follow-up.

To the best of our knowledge, this is the first study investigating the longitudinal trajectory of NSCLC from both the radiomic and liquid biopsy points of view. As far as we know, the parallelism between the dynamics of EGFR mutation status and radiomic features is potentially dependent on the progressive enrichment of tumor tissue by treatment-resistant clones.

## Conclusion

Radiomic signatures may represent a clinically relevant readout of EGFR mutational status and provide a non-invasive biomarker to monitor targeted drug therapies in NSCLC. Indeed, with the availability of big data and cutting-edge analysis strategies (such as machine learning), the information coming from tumor genotype and phenotype decoded via imaging (29), may predict treatment failures suggesting a change in treatment strategy earlier than with conventional methods. Nevertheless, it should be noted that such techniques will not substitute tissue biopsy in the near future, since they will require the aid of other parameters to be correctly interpreted and acted upon (52).

## Data Availability Statement

The raw data supporting the conclusions of this article will be made available by the authors, without undue reservation.

## Ethics Statement

The studies involving human participants were reviewed and approved by the institutional ethics committee of University Hospital, Pisa, Italy (protocol 5625/2015). The patients/participants provided their written informed consent to participate in this study. Written informed consent was obtained from the individual(s) for the publication of any potentially identifiable images or data included in this article.

## Author Contributions

Conception and design: RD, MDR, FC. Provision of study material or patients: IP, CR, SV, ML, AC, SC, ER, FC, AD, AR, RD. Collection and assembly of data: MDR, FC, SV, CR, IP, ML, SC, ER. Data analysis and interpretation: MDR, FC, CR, SC. Manuscript writing and final approval of manuscript: all authors. Financial support: RD. All authors contributed to the article and approved the submitted version.

## Funding

The authors declare that this study received funding from Astra Zeneca. The funder was not involved in the study design, collection, analysis, interpretation of data, the writing of this article or the decision to submit it for publication.

## Conflict of Interest

MDR received speaker honoraria from Astellas, Astra Zeneca, Celgene, Novartis, Pfizer, Bio-Rad Janssen-Cilag, Sanofi-Aventis; consulting fee from Ipsen and Janssen-Cilag; Speaker’s bureau: Celgene, Janssen, Sanofi; travel support from Janssen, Bio-Rad. RD received honoraria for scientific advisory board and consulting relationship from Ipsen, Novartis, Pfizer, Sanofi Genzyme, AstraZeneca, Janssen, Gilead, Lilly, Gilead, EUSA Pharma; travel support from Ipsen, Sanofi Genzyme. ML was a previous employee at Lilly and received travel support from M.S.D.

The remaining authors declare that the research was conducted in the absence of any commercial or financial relationships that could be construed as a potential conflict of interest.

## Abbreviations

AIC, Akaike’s Information Criteria; AUC, area under the curve of the ROC; CT, computed tomography; cfDNA, cell-free DNA; ddPCR, digital droplet polymerase chain reaction; DNA, deoxyribonucleic acid; ECOG, Eastern Cooperative Oncology Group; EGFR, epidermal growth factor receptor; ex19del, exon 19 deletion; GLCM, gray level co-occurrence matrix; GLNUr, gray-level non-uniformity for run; GLNUz, gray-level non-uniformity for zone; GLRLM, gray-level run length matrix; GLZLM, gray-level zone length matrix; HGRE, high gray-level run emphasis; HGZE, high gray-level zone emphasis; KV, kilovolt; LASSO, least absolute shrinkage and selection operator; LGRE, low gray-level run emphasis; LGZE, low gray-level zone emphasis; LRE, long-run emphasis; LRHGE, long-run high gray-level emphasis; LRLGE, long-run low gray-level emphasis; LZE, long-zone emphasis; LZHGE, long-zone high gray-level emphasis; LZLGE, long-zone low gray-level emphasis; mAs, milliampere-seconds; min, minutes; ml, milliliter; mm, millimeter; n, number; NGLDM, neighborhood gray-level different matrix; NSCLC, non-small cell lung cancer; PD, progressive disease; PFS, progression-free survival; PR, partial response; rpm, revolutions per minute; RLNU, run length non-uniformity; ROC, receiver operating characteristic; RP, run percentage; RTKs, receptor tyrosine kinases; SCC, squamous cell cancer; SCLC, small cell lung cancer; SD, stable disease; SRE, short-run emphasis; SRHGE, short-run high gray-level emphasis; SRLGE, short-run low gray-level emphasis; STD, standard deviation; SZE, short-zone emphasis; SZHGE, short-zone high gray-level emphasis; SZLGE, short-zone low gray-level emphasis; TKI, tyrosine kinase inhibitor; ZLNU, zone length non-uniformity; ZP, zone percentage.
