# Supplementary material for: Integrating Liquid Biopsy and Radiomics to Monitor Clonal Heterogeneity of EGFR-Positive Non-Small Cell Lung Cancer
Source: Front Oncol. 2020 Dec 16;10:593831. doi: 10.3389/fonc.2020.593831 (PMC7819134; doi:10.3389/fonc.2020.593831)
Supplement: Supplementary file 1 [file Table_1.docx]

| Sphericity | Sphericity (= 1-eccentricity) is estimated from the ratio between the volume of the ellipsoid that could describe tumor and the volume of the spheroid. Sphericity (or better, eccentricity) describes how much tumor spread varies in space (i.e. whether or not it prefers one direction over the other). The lower the eccentricity, the greater the probability that the tumor grows in the 3 directions (x-y-z), rather than favouring one. |
| --- | --- |
| Compacity | Compacity reflects how compact the volume of interest is |
| Energy | A characteristics that describes the order status of the system. Also named “Second Angular Moment” and referred to the uniformity of the gray level between voxel pairs. |
| Long-Run Low Gray-level Emphasis (SRLGE) | Short-Run Low Gray-level Emphasis is the distribution of the long homogeneous runs with low or high grey-levels. |
|  |  |
| Short-Zone Emphasis (SZE) | Short-Zone Emphasis is the distribution of the short homogeneous zones in an image. |
| High Gray-level zone emphasis (HGZE) | High Gray-level zone emphasis is the distribution of the high grey-level zones |
| Short-Zone High Gray-level Emphasis (SZLGE) | Short-Zone High Gray-level Emphasis is the distribution of the short homogeneous zones with low grey-levels. |
| Gray-Level Non-Uniformity for zone (GLNUz) | Gray-Level Non-Uniformity for zone is the non-uniformity of the grey-levels or the length of the homogeneous zones |

**Table 1. Radiomic features evaluated in the study**
