# Supplementary material for: Integrating Liquid Biopsy and Radiomics to Monitor Clonal Heterogeneity of EGFR-Positive Non-Small Cell Lung Cancer
Source: Front Oncol. 2020 Dec 16;10:593831. doi: 10.3389/fonc.2020.593831 (PMC7819134; doi:10.3389/fonc.2020.593831)
Supplement: Supplementary file 2 [file Table_2.docx]

**Table 2. Characteristics of patients**

| Number of Patients | 7 |
| --- | --- |
| Age | 57.57 ± 16.41 |
| Sex |  |
| Male | 2 (28.58) |
| Female | 5 (71.42) |
| Smoking history |  |
| Former | 3 (42.84) |
| Never | 4 (57.16) |
| ECOG |  |
| 0 | 5 (71.42) |
| 1 | 2 (28.58) |
| Histological Type at diagnosis |  |
| SCC | 1 (14.29) |
| Adenocarcinoma | 6 (85.71) |
| Stage |  |
| IV | 7 (100) |
| IIIb | 0 (0) |
| Prior EGFR-TKIs |  |
| Afatinib | 3 (42.84) |
| Gefitinib | 2 (28.58) |
| Erlotinib | 2 (28.58) |
| EGFR activating mutation at baseline |  |
| ex19del | 5 (71.42) |
| L858R | 2 (28.58) |

Data are presented as mean ± STD or n (%).

n, number; STD, standard deviation; ECOG, Eastern Cooperative Oncology Group; EGFR, epidermal growth factor receptor; TKI, tyrosine kinase inhibitor; SCC, squamous cell carcinoma.
