# Supplementary material for: Integrating Liquid Biopsy and Radiomics to Monitor Clonal Heterogeneity of EGFR-Positive Non-Small Cell Lung Cancer
Source: Front Oncol. 2020 Dec 16;10:593831. doi: 10.3389/fonc.2020.593831 (PMC7819134; doi:10.3389/fonc.2020.593831)
Supplement: Supplementary file 3 [file Table_3.docx]

**Table 3. Correlations between radiomic features and liquid biopsy**

|  |  | ex19del/L858R (copies/ml) | | T790M (copies/ml) | | C797S (copies/ml) | | Total (copies/ml) | |
| --- | --- | --- | --- | --- | --- | --- | --- | --- | --- |
|  |  | Kendall’s τb | p | Kendall’s τb | p | Kendall’s τb | p | Kendall’s τb | p |
| Histogram features | **Skewness** | -0.063 |  | 0.085 |  | 0.025 |  | -0.022 |  |
|  | **Kurtosis** | 0 |  | -0.113 |  | -0.06 |  | -0.037 |  |
| Shape features | **Volume (ml)** | 0 |  | -0.09 |  | -0.147 |  | -0.039 |  |
|  | **Sphericity** | 0.029 |  | -0.481* | 0.011 | -0.241 |  | -0.287* | 0.037 |
|  | **Compacity** | 0.069 |  | -0.085 |  | -0.232 |  | 0.008 |  |
| GLCM features | **Homogeneity** | 0.054 |  | -0.269 |  | -0.059 |  | -0.041 |  |
|  | **Energy** | 0.394* | 0.042 | -0.176 |  | -0.038 |  | 0.298* | 0.03 |
|  | **Contrast** | 0.387* | 0.046 | 0.376 |  | -0.026 |  | 0.401* | 0.038 |
|  | **Correlation** | 0.198 |  | 0.044 |  | -0.21 |  | 0.146 |  |
|  | **Entropy** | 0.052 |  | 0.292 |  | 0.04 |  | 0.127 |  |
|  | **Dissimilarity** | 0.306 |  | 0.384* | 0.048 | 0.019 |  | 0.346 |  |
| GLRLM features | **SRE** | -0.127 |  | 0.252 |  | 0.097 |  | -0.016 |  |
|  | **LRE** | 0.165 |  | -0.217 |  | -0.094 |  | 0.056 |  |
|  | **LGRE** | 0.496** | 0.009 | 0.516** | 0.006 | -0.05 |  | 0.522** | 0.005 |
|  | **HGRE** | -0.285 |  | -0.324 |  | 0.01 |  | -0.31 |  |
|  | **SRLGE** | 0.494** | 0.009 | 0.516** | 0.006 | -0.049 |  | 0.522** | 0.005 |
|  | **SRHGE** | -0.4* | 0.039 | -0.234 |  | 0.061 |  | -0.368 |  |
|  | **LRLGE** | 0.499** | 0.008 | 0.514** | 0.006 | -0.052 |  | 0.525** | 0.005 |
|  | **LRHGE** | 0.084 |  | -0.272 |  | -0.076 |  | -0.02 |  |
|  | **GLNUr** | -0.004 |  | -0.418* | 0.03 | -0.137 |  | -0.053 |  |
|  | **RLNU** | -0.014 |  | -0.407* | 0.035 | -0.162 |  | -0.048 |  |
|  | **RP** | -0.145 |  | 0.246 |  | 0.104 |  | -0.032 |  |
| NGLDM features | **Coarseness** | -0.029 |  | 0.084 |  | 0.183 |  | 0.018 |  |
|  | **Contrast** | 0.265 |  | 0.208 |  | -0.023 |  | 0.26 |  |
|  | **Busyness** | -0.073 |  | -0.156 |  | -0.163 |  | -0.115 |  |
| GLZLM features | **SZE** | 0.345 |  | 0.223 |  | 0.048 |  | 0.332 |  |
|  | **LZE** | 0.055 |  | -0.111 |  | -0.107 |  | 0.001 |  |
|  | **LGZE** | 0.52** | 0.005 | 0.508** | 0.007 | -0.055 |  | 0.538** | 0.004 |
|  | **HGZE** | -0.412* | 0.033 | -0.26 |  | 0.027 |  | -0.387* | 0.046 |
|  | **SZLGE** | 0.524** | 0.005 | 0.503** | 0.008 | -0.055 |  | 0.54** | 0.004 |
|  | **SZHGE** | -0.309 |  | -0.194 |  | 0.071 |  | -0.286 |  |
|  | **LZLGE** | 0.056 |  | -0.107 |  | -0.108 |  | 0.003 |  |
|  | **LZHGE** | 0.057 |  | -0.111 |  | -0.107 |  | 0.002 |  |
|  | **GLNU** | -0.388* | 0.046 | -0.135 |  | -0.161 |  | -0.12 |  |
|  | **ZLNU** | 0.139 |  | 0.095 |  | -0.168 |  | 0.119 |  |
|  | **ZP** | 0.119 |  | 0.332 |  | 0.122 |  | 0.197 |  |

A Kendall's correlation coefficient (tau-b, τb) was performed to evaluate any correlation between changes in radiomic features (rows) and the dynamics of EGFR mutations (copies/ml) from liquid biopsy (columns), over time. The label total copies/ml means the total number of mutant alleles. * p < .05, ** p < .01, *** p < .001.
